# Scleral Buckling: A Review of Clinical Aspects and Current Concepts

**DOI:** 10.3390/jcm11020314

**Published:** 2022-01-09

**Authors:** Matteo Fallico, Pietro Alosi, Michele Reibaldi, Antonio Longo, Vincenza Bonfiglio, Teresio Avitabile, Andrea Russo

**Affiliations:** 1 Department of Ophthalmology, University of Catania, 95100 Catania, Italy; matteofallico@hotmail.com (M.F.); pietrooliveri@hotmail.it (P.A.); antlongo@unict.it (A.L.); t.avitabile@unict.it (T.A.); andrearusso2000@hotmail.com (A.R.); 2 Department of Surgical Sciences, Eye Clinic Section, University of Turin, 10122 Turin, Italy; 3 Department of Experimental Biomedicine and Clinical Neuroscience, Ophthalmology Section, University of Palermo, 90127 Palermo, Italy; enzabonfiglio@gmail.com

**Keywords:** scleral buckling, vitreoretinal surgery, retinal detachment, encircling band, surgical complications

## Abstract

Scleral buckling represents a valuable treatment option for rhegmatogenous retinal detachment repair. The surgery is based on two main principles: the closure of retinal breaks and the creation of a long-lasting chorioretinal adhesion. Buckles are placed onto the sclera with the purpose of sealing retinal breaks. Cryopexy is usually performed to ensure a long-lasting chorioretinal adhesion. Clinical outcomes of scleral buckling have been shown to be more favorable in phakic eyes with uncomplicated or medium complexity retinal detachment, yielding better anatomical and functional results compared with vitrectomy. Several complications have been described following scleral buckling surgery, some of which are sight-threatening. Expertise in indirect ophthalmoscopy is required to perform this type of surgery. A great experience is necessary to prevent complications and to deal with them. The use of scleral buckling surgery has declined over the years due to increasing interest in vitrectomy. Lack of confidence in indirect ophthalmoscopy and difficulties in teaching this surgery have contributed to limiting its diffusion among young ophthalmologists. The aim of this review is to provide a comprehensive guide on technical and clinical aspects of scleral buckling, focusing also on complications and their management.

## 1. Surgery Overview: Historical Insight and Technical Aspects

Rhegmatogenous retinal detachment (RRD) surgery mainly includes three different surgical approaches, namely pneumoretinopexy, scleral buckling (SB) and pars plana vitrectomy (PPV). Scleral buckling is defined as the *ab esterno* approach because the surgery consists of interventions onto the scleral wall, without removing the vitreous gel.

In the first decades of the 20th century, Gonin first understood that the sealing of retinal breaks was the key principle of RRD repair [1]. Then, in the 1950s, Custodis developed a polyviol explant used to buckle the sclera [2] and Schepens described scleral buckling surgery using a polyethylene encircling tube [3]. The introduction of this type of scleral buckling surgery represented a milestone in RRD management, being a successful technique that had remained the most important treatment until the advent of pars plana vitrectomy. Thereafter, much effort has been put into improving vitrectomy techniques until the introduction of minimally invasive pars plana vitrectomy. Historically, vitrectomy has gained ever-increasing popularity in the last three decades, being preferred over scleral buckling by most surgeons [4,5]. A possible reason for this shift from scleral buckling towards vitrectomy could be also related to the fact that interest in scleral buckling has slightly declined over the time, due to difficulties in both teaching and learning this surgery, which depends on great experience in binocular indirect ophthalmoscopy and scleral indentation. Nowadays, fellows and young surgeons usually have more confidence in slit lamp-based ophthalmoscopy rather than in binocular indirect ophthalmoscopy; vitreoretinal training tends to teach trainees to perform vitrectomy steps early, with the result of gaining more confidence and experience in vitrectomy. However, scleral buckling continues to have a relevant role in clinical practice and it should be the treatment of choice for specific types of RRDs, ensuring better visual and anatomical outcomes compared to pars plana vitrectomy.

Basically, scleral buckling surgery is based on two main principles, which allow the reattachment of the retina. One is the closure of retinal breaks and the reduction/elimination of vitreoretinal tractions. The other is to ensure a proper and long-lasting chorioretinal adhesion. Retinal break closure and vitreoretinal traction reduction are obtained by suturing buckling elements, called explants, onto the scleral surface. Retinopexy is performed to make chorioretinal adhesion stronger and longer-lasting. This surgery relies on the correct localization of all retinal breaks, which depends on a thorough fundus examination by using a binocular indirect ophthalmoscope with scleral indentation. All retinal breaks need to be sealed properly: retinal break borders must lay inside the buckling area. Buckle type and size need to be chosen carefully. Either a non-properly sealed break or a missed tear can cause a failure of the surgery. Usually, the explants are in silicone material, either solid or sponge. The explants are sutured onto the scleral surface most often through 5-0 non absorbable mattress sutures. The explants could be classified into the radial buckle and circumferential buckle; the latter one could be segmental or encircling, that is, extending for 360 degrees. Radial buckles are placed radially to the limbus. Usually, silicone sponges are used for radial buckles. This type of buckle is useful in the case of a single retinal break, adjacent breaks within 1 clock hour, or posterior breaks. The advantage of a radial silicon sponge is to provide an excellent closure of the tear. However, if the tear is too posterior, there is the risk of retinal distortion which could affect the macula and might require the buckle to be removed. A 360 circumferential buckle consists of an encircling silicone band, with 2 to 4 mm height. The advantage of using an encircling band is to have a buckling effect for 360 degrees, which helps to reduce vitreo-retinal tractions and to treat multiple breaks and peripheral degenerative areas. In particular, the band is placed posteriorly to the vitreous base: the anterior edge of the encircling buckle should run along the posterior edge of the vitreous base. The vitreous base is where most likely degenerative processes happen and usually the anterior border of retinal breaks is in proximity to the vitreous base. The encircling band is meant to create a sort of new *ora serrata*, a safety area which is supposed to protect from the vitreous base’s degenerative processes. The encircling band is placed in proximity to the equator (or anteriorly), at 11–14 mm from the limbus. The band runs underneath the four recti muscles and is secured to the sclera through non absorbable sutures. The encircling band is usually closed through a silicone sleeve: its closure represents one of the final steps of the surgery because it leads to an intraocular pressure rise. Segmental circumferential buckles, such as strips and tires, are usually placed underneath the encircling band at retinal breaks’ locations, with the purpose of sealing the breaks and reducing the risk of the fish-mouthing of a retinal tear. Fish-mouthing is a possible drawback of the encircling band: the encircling buckle may determine a redundant retina with subsequent folds, which could cause this phenomenon if involving a retinal break. When there is fish-mouthing of a retinal tear, the use of a radial sponge may help solve it and seal the tear properly. Traditional scleral buckling surgery usually involves: conjunctival peritomy; recti muscles isolation; localization of each retinal break; encircling band application and buckles positioning to seal each break properly; accurate retinopexy to all breaks (can be based on either cryotherapy –more commonly- or laser photocoagulation); an additional step performed by most surgeons is the evacuative puncture. The evacuative puncture allows subretinal fluid to be drained intraoperatively. Advantages of this procedure are: a better buckling effect of the explants; a better effect of the retinopexy; the possibility to evaluate whether the buckles are positioned correctly with all tears sealed. The evacuative puncture is useful in the case of bullous detachment or macula-off detachment. However, this procedure is associated with a high risk of complications, such as: bleeding (e.g., choroidal/subretinal/retinal hemorrhages which can threaten the visual outcome and, in some cases, cause visual loss); vitreous/retinal incarceration; retinal tear entrapment; retinal perforation; and hypotony. Evacuative puncture should be performed far from the choroidal vortex vein and long ciliary arteries, and far from retinopexy areas. Ocular hypotony subsequent to the puncture could be dealt with by tightening the encircling band and injecting intravitreal air or gas bubbles. Even if most surgeons tend to perform this step, it could be unnecessary in the case of RRDs localized to a single quadrant and with small amount of subretinal fluid. Indeed, it is important to point out that if each retinal break is properly closed, the subretinal fluid is likely to be drained by a retinal pigment epithelium pump. On this basis, an alternative and less invasive scleral buckling procedure was first introduced by Lincoff in 1965 [6] and, then, refinished by Kreissig [7]. This technique, called minimal segmental buckling without drainage or minimal extraocular surgery, involves the application of buckle and cryopexy to each break, neither applying an encircling band nor the evacuative puncture. According to Kreissig, 90% of RRDs can be treated with minimal segmental buckling without drainage [7]. Furthermore, Lincoff and Kreissig provided useful guidelines for localizing retinal breaks in RRD: four rules for identifying primary retinal breaks and four rules for identifying missed retinal breaks after unsuccessful surgery [7,8,9].

With regards to retinopexy, Lincoff was the first to use cryopexy for the treatment of retinal tears. Retinopexy represents an essential step of scleral buckling surgery. However, some authors suggest not performing retinopexy [10,11] while others support a deferred retinopexy [12]. Usually, retinopexy is performed either before or after the evacuative puncture during the scleral buckling procedure [13] and, in general, after buckles placement. Nowadays, retinopexy could be achieved through laser photocoagulation or cryotherapy (cryopexy), with no difference in terms of anatomical outcomes between the two procedures [14].

Each step of the SB procedure has a noticeable influence on the outcome. A proper closure of all retinal breaks is necessary to achieve anatomical success. Indirect ophthalmoscopy with scleral indentation has a key role in identifying retinal breaks, allowing their correct localization onto the sclera. A thorough preoperative examination of the patient is mandatory to evaluate RRD characteristics and to identify all retinal breaks. An ancillary test that can be useful both for preoperative assessment and postoperative follow-up is the ultra-wide field imaging. Ultra-wide field retinal imaging can provide precise information on RRD extent and causative breaks [15]. However, its sensitivity in detecting lesions in the inferior and superior periphery is limited and steered imaging of those areas might be required [15]. Ultra-wide field retinal imaging can be considered a useful adjunct to clinical fundus examination.

**Figure 1:**
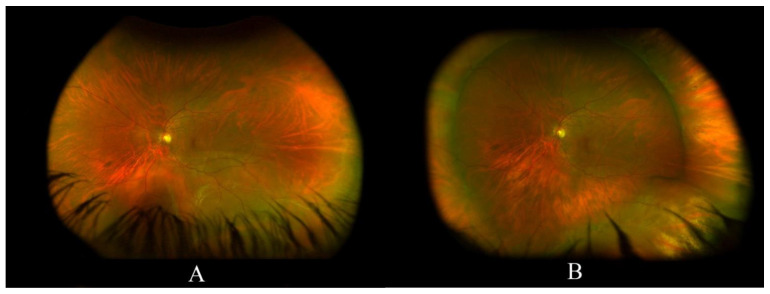
(**A**) a left eye inferior rhegmatogenous retinal detachment in a young phakic patient, the macula looks attached; (**B**) a fully reattached retina following scleral buckling with a 360 encircling band with an inferotemporal buckle.

Figure 1 illustrates a case of an inferior RRD successfully treated with SB.

## 2. Clinical Indications

When it comes to which types of RRDs are most suitable for scleral buckling, and, as a consequence, when this procedure should be preferred over vitrectomy, the results of the Scleral Buckling versus Primary Vitrectomy in Rhegmatogenous Retinal Detachment (SPR) study provide information [16]. The SPR study is a large randomized clinical trial that enrolled more than 500 eyes and compared scleral buckling versus vitrectomy in the management of medium complexity RRD. Medium complexity RRD included: RRD with large breaks (1–2 clock hours’ size); RRD with marked vitreous tractions; RRD with multiple breaks; RRD with central extension of the break; superior bullous RRD; RRD with different anterior-posterior localization of breaks [17]. A proliferative vitreoretinopathy (PVR) grade B and C was considered amongst the exclusion criteria [17]. The results of this trial showed that in phakic eyes, scleral buckling yielded a better visual outcome compared with vitrectomy, while there was no difference in terms of anatomical success and postoperative PVR (grade B–C); additionally, in phakic eyes, scleral buckling was associated with a lower rate of postoperative cataract development compared with vitrectomy (46% versus 77%) [16]. In the pseudophakic cohort, the rate of primary anatomical success was significantly higher in eyes treated with vitrectomy compared with scleral buckling (72% versus 53%), while no difference was found between the two procedures with regards to visual outcome and postoperative PVR (grade B–C) [17]. As a result, scleral buckling should be preferred over vitrectomy in phakic eyes with medium complexity RRD, given the better visual outcome and the lower incidence of postoperative cataract; vitrectomy should be preferred over scleral buckling in pseudophakic eyes with medium complexity RRD, given the higher rate of primary anatomical success provided by PPV. Thereafter, a multicenter study from the European Vitreo-Retinal Society retrospectively reviewed outcomes of more than 7000 cases with uncomplicated RRD, that is with no greater than C-1 grade PVR [18]. This report showed that in phakic eyes with uncomplicated RRD, scleral buckling yielded a lower final failure rate compared with vitrectomy; in the pseudophakic cohort, the percentage of eyes with surgery failure after the initial procedure was lower for vitrectomy than scleral buckling [18]. These findings are in line with those reported by the SPR study. Accordingly, scleral buckling represents a valuable treatment option in phakic eyes with uncomplicated RRD or medium complexity RRD. Of note, simple RRDs in phakic eyes, with a single break (or small cluster of breaks within one clock hour) in the superior eight clock hours should be managed, if possible, with pneumatic retinopexy [19]. In general, surgeons should go for the treatment option that is most suited to RRD features, with greater odds of better outcomes, and is, at the same time, less invasive.

Very recently, the results of the Primary Retinal Detachment Outcomes Study have been published [20,21]. This is a retrospective multicenter study that gathered data on outcomes of primary RRD repair surgery from five large institutes in the United States, publishing two reports according to the lens status, that is, phakic [21] and pseudophakic [20]. The report on phakic RRD included eyes compared three different interventions: scleral buckling, vitrectomy alone and vitrectomy combined with buckling [21]. Eyes with ‘moderately complex RRD’ were enrolled. This definition was principally based on the SPR study. The authors excluded those cases presenting clinical characteristics that could strongly influence the choice of treatment towards either scleral buckling or vitrectomy (with or without additional buckle). Therefore, moderate to dense vitreous hemorrhage, any PVR, previous vitrectomy, vitreous opacities, giant tear, significant cataract, prior glaucoma surgery, planned internal limiting membrane peeling, age < 40 years, detachment extent either < 3 h or >9 h, were considered as exclusion criteria. This report included 715 eyes: 169 scleral buckling cases, 249 vitrectomy cases, 297 combined procedure cases. Scleral buckling was found superior to vitrectomy alone and combined with buckling for final anatomic success (99.4% for scleral buckling, 96.3% and 96.6% for vitrectomy alone and combined with buckling, respectively). The rate of single surgery anatomic success was 91.7% for scleral buckling, significantly higher compared to vitrectomy alone (83.1%). The best visual outcome was shown in the scleral buckling group, significantly greater than vitrectomy alone or combined with buckling [21]. These findings are in line with those reported by the SPR study and the European Vitreo-Retinal Society report, corroborating the fact that scleral buckling could provide better outcomes compared with vitrectomy in phakic eyes with uncomplicated or moderately complicated RRDs.

The Primary Retinal Detachment Outcomes Study report on pseudophakic RRDs compared vitrectomy alone versus vitrectomy combined with scleral buckling, including a total of 893 eyes, 684 vitrectomy cases and 209 combined cases. Vitrectomy combined with scleral buckling yielded a better single surgery anatomic success rate compared with vitrectomy alone, 92% versus 84%, respectively. However, no difference in final visual outcome was found between the two groups [20].

The issue as to whether vitrectomy combined with scleral buckling could provide better outcomes compared with vitrectomy alone has been long debated. The above-mentioned report from the Primary Retinal Detachment Outcomes Study showed a higher single surgery anatomic success for the combined surgery in cases with pseudophakic RRDs, but with no difference in final visual outcome [20]. A combined approach could be useful, in particular, for the management of RRD with inferior break. Some authors reported a better anatomical success for a combined approach in RRD with inferior break [22,23]. This could be related to the fact that gas tamponade might not have an effective tamponade effect on inferior breaks, while a buckle could close it [23]. However, other authors showed comparable outcomes between vitrectomy alone and vitrectomy combined with scleral buckling for the management of RRD with inferior breaks [24,25]. A randomized clinical trial compared anatomical outcomes between vitrectomy combined with an encircling band versus vitrectomy alone. Subgroup analyses of pseudophakic RRDs with inferior breaks revealed a trend of better anatomical success in eyes treated with vitrectomy combined with an encircling band, but without reaching statistical significance [26]. This issue appears controversial. It is worth noting that the above-cited report from the European Vitreo-Retinal Society, which retrospectively reviewed outcomes of more than 7000 cases with uncomplicated RRD, concluded that in eyes undergoing vitrectomy, a supplemental buckle does not provide beneficial outcomes [18].

A multicenter retrospective study compared SB with vitrectomy (25 or 27 gauge) in young patients with RRD, including 295 and 262 eyes in the SB group and PPV group, respectively [27]. Primary anatomical success was comparable between the two procedures (92.2% in the SB group versus 93.9% in the PPV group), but eyes treated with SB had a better final visual outcome and lower rates of cataract formation and PVR onset [27].

Other clinical conditions that can be treated with scleral buckling include retinal detachment complicating retinoschisis [28] and retinal detachment secondary to dialysis [22].

## 3. Anatomic Success

Many variables may have an influence on anatomical outcome, such as lens status and RRD characteristics. The SPR study demonstrated that primary anatomical success in phakic eyes with medium complexity RRD was achieved in 63.6% of cases treated with SB, while final anatomical success was reached in 96.7% [16]. In pseudophakic eyes with medium complexity RRD, primary anatomical success was obtained in 53.4% and final anatomical success in 93.2% [16]. The EVRS study reported a total failure rate following SB for uncomplicated RRD of 15.2% and 24.8% in phakic and pseudophakic eyes, respectively [18]. According to the Primary Retinal Detachment Outcomes Study, phakic eyes with a moderately complex RRD showed a 91.7% rate of single surgery anatomic success following SB, while final anatomic success was obtained in 99.4% of cases [21]. A recent study demonstrated a 95% success rate following SB for uncomplicated phakic macula on retinal detachment [29]. The main reason for surgical failure after SB procedure remains proliferative vitreoretinopathy (PVR) development: in a study of more than 500 eyes treated with SB for primary RRD, re-operation rate amounted to 13%, surgical failure being secondary to PVR formation in 5% of cases [30]. Moreover, some authors described the absence of pre-operative posterior vitreous detachment (PVD) as another cause of surgical failure in SB procedure, because the evolution of PVD after SB could lead to new retinal tears [22] or could be associated with PVR [31].

## 4. Complications

Scleral buckling complications can be classified as intra-operative and post-operative.

### 4.1. Intra-Operative Complications

#### 4.1.1. Anesthesia-Related Complications

Complications secondary to local anesthesia (peribulbar or retrobulbar) are rare but very serious. These include retrobulbar hemorrhage, penetration or perforation of the globe, optic neuropathy, diplopia secondary to muscle injury and respiratory arrest because of dural sheath inoculation with subsequent brainstem anesthesia [32,33]. If an increase in intraocular pressure is suspected, an immediate observation of the central retinal artery with an indirect ophthalmoscope and a paracentesis of the anterior chamber should be performed [34]. Control of hemodynamics is necessary in the case of retrobulbar hemorrhage to preserve and limit optic nerve injury; canthotomy could be necessary in the case of severe retrobulbar hemorrhages [34].

#### 4.1.2. Complications Occurring during Subretinal Fluid Drainage

This step has the greatest potential for complications. Draining subretinal fluid is still controversial. Anatomical and functional results proved to be the same with or without drainage, but complication rate was greater in the drainage group [35]. On the other hand, subretinal fluid drainage is still necessary when the indentation of the buckle element alone is not appropriate for closing the retinal break [34]. Many variables have an influence on the location of the drainage, such as RRD characteristics and the distribution of subretinal fluid, location of retinal breaks, vitreoretinal and epiretinal membrane tractions, configuration of buckle elements, and the choroidal vascular network. A good strategy in order to reduce the risk of retinal damage is to perform the evacuative puncture far enough from retinal breaks and where the retina is the most highly detached [34]. Complications related to the drainage procedure include choroidal/subretinal/retinal hemorrhages, vitreous/retinal incarceration, retinal tear entrapment, retinal perforation, hypotony. In the case of retinal incarceration, a buckle should be placed at the puncture site and cryotherapy is necessary [34]. As the residual SRF drains, the retina gets flattened against the retinal pigment epithelium [36].

#### 4.1.3. Scleral Rupture

This complication has been reported in 5% of the cases [37]. It is often associated with preexisting scleral thinning due to high myopia or preexisting scleral pathologies. During the scleral buckling procedure, the sclera is exposed to locate retinal breaks and to place sutures and buckles. A full and thorough inspection of the sclera is required in order to mark scleral thinning and to plan the most appropriate buckle approach [38]. If the sclera is too thin to hold a buckle or a suture, consider not suturing the buckle in that quadrant, even more if that area does not need a buckle to specifically support a break. In some occasions, when the sclera is too weak, it could be necessary to switch to vitrectomy [34]. If a scleral perforation occurs where the retina is detached, this typically leads to drainage of the subretinal fluid; if the retina is attached, it can lead to retinal perforations and hemorrhage [37]. When the surgical procedure is performed in eyes with previous retinal detachment surgery, scleral depression over weak areas, such as suture site, cut-down sclerotomies or scleral explant site, could cause a frank scleral rupture. Tissue glue could be used to restore the globe integrity. Scleral rupture can lead to secondary intraoperative complications such as retinal incarceration and subretinal, choroidal or vitreous hemorrhages [37].

#### 4.1.4. Scleral Perforation

If an inadvertent scleral perforation occurs during sutures placement and a massive release of subretinal fluid happens, the management of surgical procedure can be challenging for the surgeon even if subretinal fluid drainage has been planned [39]. Scleral perforation can be associated with a subretinal or sub-choroidal hemorrhage, which could be self- limiting; when the hemorrhage tends to extend toward the posterior pole, air or gas tamponade and prone position are recommended. Scleral perforation could be associated with severe ocular hypotony. In the case of scleral perforation, it is necessary to limit the loss of intraocular contents and to restore intraocular pressure. A wide stitch could be placed above the accidental site of drainage so that subsequent tightening would seal off the drainage site. If a vitreous prolapse occurs, gently cut it off with scissors. A patch graft and/or a silicone explant can be also placed above the perforation site; in particular, this could help in the case of retinal incarceration [37]. Tissue glue can be used but it requires a dry surgical field. Intravitreal injection of gas or air could be necessary to restore intraocular pressure [40].

#### 4.1.5. Hypotony

Ocular hypotony could be secondary to evacuative puncture or scleral perforation, as mentioned above. Complications resulting from severe hypotony include retinal incarceration and vitreous, choroidal and/or subretinal hemorrhages. These complications lead to a series of difficulties during and after surgery (such as hemorrhages interfering with visualization and distortion of the globe) up to jeopardizing the final outcomes. The management of hypotony depends on its severity. In the case of mild to moderate hypotony, encircling band tightening could help to restore intraocular pressure. In the case of severe hypotony, intraocular injection of an air/gas bubble could be performed as well. In the case of scleral perforation, it is important to deal with it as described in the previous paragraph.

#### 4.1.6. Choroidal Detachment

Choroidal detachment is one of the most common complications after scleral buckling, occurring in 23–44% of cases [41,42]. It is usually observed in the first days postoperatively, but in some cases it can occur intraoperatively or immediately after surgery [43]. The precise pathogenesis has not been completely understood yet. In most cases this complication is self-limiting and tends to settle down spontaneously in about 2 weeks [44]. Choroidal detachment can be classified as hemorrhagic, serous and sero-hemorrhagic. Many variables can have an influence on this complication:

- Preoperative: advanced age (aging can affect choroidal vasculature), myopia, and systemic conditions (such as hypertension) [44];
- Intraoperative: extension of the buckle, intraocular pressure fluctuation (hypotony), injury to a vortex vein (an imbalance between the pressure into the choroidal vascular network and low intraocular pressure is assumed to be the trigger for choroidal detachment development) [43,44].

Depending on the severity of the condition, its management could be either medical or surgical. It is necessary to deal with intraocular pressure in order to obtain normal values. Topical and systemic steroids need to be administered immediately after the surgery. Diuretic drugs can be useful for obtaining a rapid absorption of suprachoroidal fluid [44]. In more complex cases, a surgical drainage of suprachoroidal hemorrhage is advocated [45]. Surgical management of suprachoroidal hemorrhage can be a primary drainage alone, a secondary drainage alone, or both primary and secondary drainage. Indications for primary drainage include: a massive intraoperative hemorrhage and/or inability to reposit intraocular contents. A posterior sclerotomy is performed intraoperatively. Secondary drainage represents a delayed approach. This is performed when there is evidence on ultrasound of suprachoroidal hemorrhage liquefaction [45]. A posterior sclerotomy is performed under a constant fluid infusion at limbus. Suprachoroidal hemorrhage drainage could be associated with vitrectomy [46].

#### 4.1.7. Subretinal and Intravitreal Hemorrhage

Subretinal hemorrhage is a sight-threatening complication that can occur as a result of a deep suture, following scleral depression, or during subretinal fluid drainage [47]. The visual outcome depends on whether the bleeding reaches the macula or not. Self-limiting and small hemorrhages not reaching the macula commonly do not affect visual and anatomical outcomes. When the blood collects in the submacular area, it can cause severe visual impairment [47]. Subretinal blood can cause damage to photoreceptors in several ways: a barrier effect mechanism between photoreceptor and retinal pigment epithelium; a toxic effect caused by iron; and/or mechanical injury caused by coated blood [47]. Subretinal blood can impede retinal reattachment and promote PVR [47]. Management of subretinal bleeding depends on its severity. Small and self-limiting hemorrhages might not require treatment and can be successfully managed by positioning the patient in order to prevent the blood from reaching the macula. In some cases, the intravitreal injection of air can be useful for this purpose. In more severe cases, subretinal drainage with an extrusion needle might be required. If vitrectomy is needed, perfluorocarbon liquids could be used to displace subretinal blood and drain it through a retinotomy or a break [47]. The use of tissue plasminogen activator (TPA) has proved useful, especially if there is a significant clot [48].

**Figure 2:**
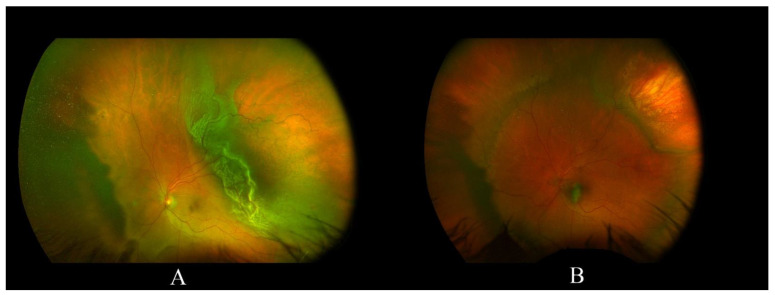
(**A**) a case of supero-temporal retinal detachment with an attached fovea; (**B**) following SB the retina is fully reattached, but a macular subretinal hemorrhage occurred.

Figure 2 shows a case of macular subretinal hemorrhage following SB.

### 4.2. Post-Operative Complications

Scleral buckling can be associated with severe postoperative complications. Surgical failure has been discussed in the ‘anatomic success rate’ paragraph. Appropriate patient selection, careful planning, and good intraoperative technique can help to limit the rate of complications and improve outcomes.

#### 4.2.1. Refractive Changes

Astigmatic or non-astigmatic refractive changes following scleral buckling are related to changes in eye-ball shape induced by the surgery. There is controversial data on the onset of astigmatism after scleral buckling, being infrequent and transient in most studies [49,50]. Risk factors for postoperative astigmatism include buckle height, the use of radial buckles, medial rectus disinsertion and anterior location of the buckle. A very common refractive change following scleral buckling is a myopic shift, secondary to an increase in the axial length [49,51]. Buckle height has an influence on the amount of induced myopia. A higher degree of induced myopia is more likely in phakic eyes due to an anterior displacement of the lens [49,51]. It seems that in children and young patients, this myopic shift is less prominent, being assumed that sclera buckling might impede ocular growth [52].

#### 4.2.2. Infection

In the past, scleral abscesses following scleral buckling were reported in about 4% of cases [53]. Over the years, the technique has been refined. Diathermy has been replaced by cryotherapy. As a result, the scleral abscess rate reduced to 0.58% [54]. The main symptoms of scleral abscess include the presence of white extrascleral lesions, pain and vitreitis. There is a high risk of perforation if the buckle is not immediately removed. Extraocular infection following scleral buckling can occur between 0.5% and 5.6% of cases [55,56,57]. Most commonly, causative micro-organisms belong to Staphylococcus species [58]. Presoaking of the buckle elements in an antibiotic solution was thought to be helpful for infection prophylaxis. However, a large retrospective study showed that presoaking does not reduce extrusion and infection, whereas both disinfection prophylaxis and accurate surgical technique are mandatory [59]. Endophthalmitis following scleral buckling surgery is extremely rare [60].

#### 4.2.3. Extrusion and Intrusion of Buckles

Buckle extrusion usually causes redness and pain. This complication represents the main reason for buckle removal: one out of two buckles that need to be removed is because of extrusion [61]. Extrusion through the skin is extremely rare. Buckle intrusion occurs in 3.8–18.6% of cases [62,63] and is more likely if intrascleral implants are used [64]. Buckle intrusion can be associated with retinal detachment, hypotony, vitreous hemorrhage and endophthalmitis. Management depends on the severity of this complication. Observation can be appropriate in asymptomatic patients. If the above-mentioned symptoms occur, the buckle needs to be removed [64]. The scleral defect can be covered by using cyanoacrylate glue, scleral imbrication or other scleral patching techniques [63,64]. Vitrectomy may be required to deal with intrusion-related complications [64].

**Figure 3:**
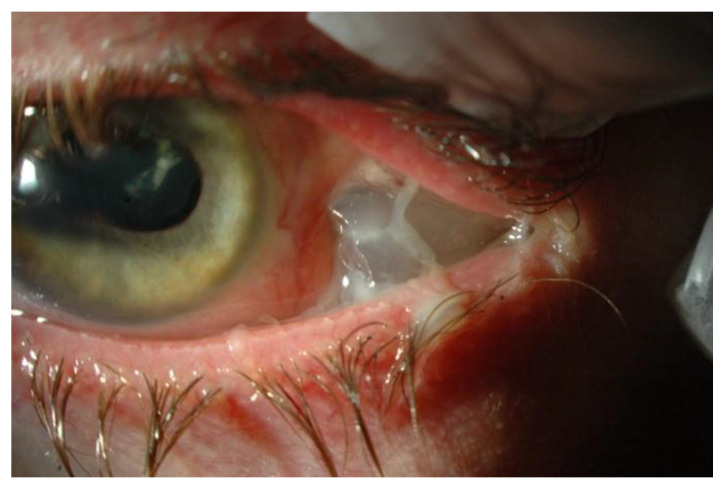
A left aphakic eye with an extrusion of a MIRAgel (hydrogel) buckle through the temporal conjunctiva.

Figure 3 shows a case of buckle extrusion through the conjunctiva.

#### 4.2.4. Anterior and Posterior Segment Ischemia

Anterior segment ischemia following scleral buckle seems to be related to vascular non-perfusion of anterior and/or posterior ciliary arteries, or alteration in retinal vasculature [65,66,67,68]. Clinical variables that may have an influence on this complication include old age and hematologic factors such as sickle cell disease and the presence of atheromatosis. Cryotherapy or buckle compression can cause an injury to the posterior ciliary arteries, leading to non-perfusion of the anterior segment [69]. Disinsertion or manipulation of recti muscles could also affect anterior segment perfusion [69]. Indications for buckle removal include anterior segment ischemia and iris neovascularization [69]. Abnormal retinal blood flow has been shown after scleral buckling with an encircling band [65,66]. Anterior segment ischemia has been found even in cases where a segmental buckle was used [67]. A posterior segment ischemia has been demonstrated following scleral buckling, but ophthalmic artery blood flow seems to be not significantly affected after the procedure [68].

#### 4.2.5. Diplopia

Double vision is a significant postoperative complication secondary to muscle imbalance issues. In a retrospective study, 3.8% of cases developed a secondary strabismus following scleral buckling [70]. The majority of cases had a mechanical muscle restriction. The position of the buckle seems to be not associated with the incidence of diplopia. A moderate association between buckle position and diplopia was shown when two muscles were involved [70]. Several causes of diplopia have been reported: the buckle itself (mechanical restriction of the extraocular muscles), local anesthetics myotoxicity [71] and extraocular muscles ischemia (injury to the muscles, especially in old patients, can cause ischemia) [70,72]. Prism correction usually represents the initial management of postoperative non-resolving diplopia. Muscle surgery with or without buckle removal might be required when symptoms are not alleviated conservatively [72].

#### 4.2.6. Cataract

The risk of cataract development has been clearly demonstrated to be higher after PPV rather than SB procedure [16,21]. The SPR study showed a progression of cataract over a 1-year follow-up in 46% of phakic eyes treated with SB and in 77% of phakic eyes treated with PPV [16]. Such a high rate of cataract progression in SB cases needs to be considered cautiously. The SPR study defined cataract progression as an increase of one grade or greater on the Lens Opacities Classification System III [16]. Furthermore, some of these patients might have developed a cataract progression because of aging. More recently, the *Primary Retinal Detachment Outcomes Study* reported a 17% rate of cataract surgery after SB over a mean follow-up longer than 1 year, much lower compared with a 66% rate after PPV [21]. Schwartz et al. [73] reviewed the outcomes of SB cases with a follow-up of 20 years or longer, reporting that cataract surgery was performed in 30% of phakic eyes on this long term period.

#### 4.2.7. Persistent Subretinal Fluid

Recently, OCT has played an important role in the diagnosis and follow-up of retinal detachment. Because of its high-resolution images, it can be used to show some details that would be very difficult to detect using standard equipment such as a slit lamp or indirect ophthalmoscopy examination. Persistent subretinal fluid (SRF) can be found with OCT and has been reported in between 27% and 78% at four to six weeks after scleral buckle surgery for retinal detachment [74]. It is still unclear if persistent subretinal fluid can influence visual outcome: several reports suggest that SRF could be associated with delayed visual recovery or poor visual outcome [74,75], but some authors proposed that residual SRF has no influence on visual recovery in the long term [76,77]. Kim et al. analyzed risk factors associated with the development of SRF in successful SB after RRD and reported that only two factors were significantly associated with 1-month postoperative SRF: preoperative macular status (macula-off) and segmental SB [78]. The use of an encircling surgery was associated with a lower incidence of SRF [78]. Other authors demonstrated that younger age and high myopia were clinical variables associated with persistent SRF following SB procedure [79]. Fu et al. identified three patterns of persistent SRF following SB surgery: bleb-like loculated (BL), shallow-diffused (SD), and multiple blebs (MB) [79]. More than 50% of SRF showed the BL pattern and this pattern was the one with shortest duration. Most cases of SD pattern transformed into the BL pattern over the follow-up time [79].

#### 4.2.8. Macular Edema and Macular Epiretinal Membrane

The incidence of cystoid macular edema (CME) following the SB procedure for RRD has been reported as between 5.6% and 43% of cases [80,81,82]. A recent study including 130 eyes found a CME incidence rate of 6.9% [83]. Older age, macular detachment and external fluid drainage have been shown to be risk factors for CME development [83]. The use of cryopexy has been assumed to be associated with postoperative inflammation, which can promote CME development [83]. However, a previous study comparing cryopexy versus transscleral diode laser retinopexy did not show a higher incidence of CME in eyes receiving cryotherapy [84]. Given the inflammatory pathogenesis, the intravitreal 0.7 mg dexamethasone implant has been used for the treatment of this condition, showing favorable outcomes [85].

Macular epiretinal membrane has been reported in about 18% of eyes following SB for primary uncomplicated retinal detachment [86]. Interestingly, it seems that development of macular epiretinal membrane is more common in phakic eyes compared with pseudophakic ones, with an incidence of this complication in 15.2% and 7.7% of cases, respectively [87]. In general, precipitating factors of epiretinal membrane following RRD repair surgery are an older age, the presence of vitreous hemorrhage, the presence of large retinal tears and intraoperative cryotherapy [88]. These factors support the hypothesis that the pathogenesis of this complication is related to the dispersion of retinal pigment epithelial cells into the vitreous chamber [88].

### 4.3. Comparison between Scleral Buckling and Vitrectomy

According to a report conducted among Medicare beneficiaries, vitrectomy complications can be classified as severe (i.e., endophthalmitis, suprachoroidal hemorrhage, PVR, rhegmatogenous retinal detachment) and less severe (i.e., choroidal detachment, vitreous hemorrhage, retinal edema, glaucoma, retinal tear, hypotony, corneal edema, corneal abrasion) [89]. Lv et al. performed a meta-analysis that compared complications between SB and PPV for rhegmatogenous retinal detached [90]. A total of six randomized trials and three retrospective studies were included. Pooled rates of surgical complications reported by the authors are shown in Table 1.

## 5. Conclusions

SB remains a valuable surgical option for RRD repair. This surgery yields better anatomical and functional outcomes in phakic eyes with uncomplicated or moderately complicated detachments. However, in recent years there has been a decline in SB procedures in favor of vitrectomy. Reports conducted among Medicare beneficiaries showed that the use of the SB procedure decreased by 69% from 1997 to 2007 [4], continuing to decline in later years: in 2009, 11% of RRD cases were treated with SB while only 5% of RRD cases underwent the SB procedure in 2014 [91]. This trend seems to be related to several factors.

First, significant improvements have been made in vitrectomy systems and modern research continues to invest in developing ever-innovative technologies. However, cost analysis studies demonstrated that SB is less expensive than vitrectomy in phakic patients [92], providing a slightly better cost-effectiveness ratio [93].

Furthermore, the operating time is shorter for vitrectomy compared with SB. On average, an SB procedure takes one hour or a bit longer to complete [94], while a vitrectomy can be performed in 45 min [95]. This could have an influence on surgical planning.

With regard to patient satisfaction, a survey reported a higher proportion of patients experiencing discomfort or pain during SB surgery compared with vitrectomy (57% versus 10%, respectively) [96]. However, the type of surgery seems to have no influence on vision related quality of life [97].

Ultimately, a relevant contributing factor to this declining trend in SB use might be a decreasing exposure to SB surgery in training programs. Young fellows tend to be trained in slit-lamp based fundoscopy, with less training in indirect binocular ophthalmoscopy, which is a *conditio sine qua non* for SB surgery. Both teaching and learning the SB procedure could prove challenging. University hospitals should strive to teach trainees indirect binocular ophthalmoscopy and the SB procedure in order to avoid this surgery becoming obsolete in the foreseeable future.

## Figures and Tables

**Table 1:** Pooled rate of surgical complications after scleral buckling and vitrectomy for rhegmatogenous retinal detachment. Data from Lv et al. 2015 [90].

Complications	Scleral Buckling	Vitrectomy
Subretinal haemorrhage	5.1%	0.9%
Hypotony	23.2%	0%
Iatrogenic breaks	0.2%	8.2%
Choroidal detachment	3.1%	0%
Residual SRF	19.6%	0%
High IOP	5.4%	11.6%
Corneal epithelial defect	1.8%	5.5%
Diplopia	2.7%	0.5%
Cataract	23.6%	53.1%
CME	2.6%	2.8%
macular pucker	7.4%	5.7%
Postoperative PVR	11.2%	11.1%

Footnote: SFR, subretinal fluid; IOP, intraocular pressure; CME, cystoid macular edema; PVR, proliferative vitreoretinopathy.

## Data Availability

Data sharing not applicable.
